# Reactivation of epigenetically silenced miR-124 reverses the epithelial-to-mesenchymal transition and inhibits invasion in endometrial cancer cells via the direct repression of IQGAP1 expression

**DOI:** 10.18632/oncotarget.7754

**Published:** 2016-02-26

**Authors:** Peixin Dong, Kei Ihira, Ying Xiong, Hidemichi Watari, Sharon J.B. Hanley, Takahiro Yamada, Masayoshi Hosaka, Masataka Kudo, Junming Yue, Noriaki Sakuragi

**Affiliations:** ^1^ Department of Women's Health Educational System, Hokkaido University School of Medicine, Hokkaido University, N15, W7, Sapporo, Japan; ^2^ Department of Gynecology, Hokkaido University School of Medicine, Hokkaido University, N15, W7, Sapporo, Japan; ^3^ Department of Gynecology, State Key Laboratory of Oncology in South China, Sun Yat-sen University Cancer Center, Guangzhou, P. R. China; ^4^ Department of Pathology and Laboratory Medicine, University of Tennessee Health Science Center, USA; Center for Cancer Research, University of Tennessee Health Science Center, Memphis, TN, USA

**Keywords:** IQGAP1, miR-124, endometrial cancer cell invasion, epigenetics, DNA methyltransferase inhibitor

## Abstract

Overexpression of IQGAP1 and microRNA (miRNA) dysregulation are frequent in human tumors, but little is known about the role of IQGAP1 and its relationship to miRNA in endometrial carcinogenesis. We demonstrate that IQGAP1 activates the epithelial–mesenchymal transition (EMT) program and that miR-124 directly represses IQGAP1 expression in endometrial cancer (EC) cells. The overexpression of IQGAP1 stimulates EMT features and enhances migration, invasion and proliferation of EC cells, whereas knocking down IQGAP1 expression reverses EMT and inhibits these malignant properties. Using miRNA microarray profiling, we identified 29 miRNAs (let-7b, let-7f, miR-10b, miR-15b, miR-23a, miR-24, miR-25, miR-27a, miR-29b, miR-30a-5p, miR-34a, miR-124, miR-127, miR-130b, miR-148a, miR-155, miR-191*, miR-194, miR-224, miR-362, miR-409-3p, miR-422b, miR-424, miR-453, miR-497, miR-518d, miR-518f*, miR-526a and miR-656) that are significantly down-regulated in an *in vitro*-selected highly invasive derivative cell line (HEC-50-HI) relative to the parental HEC-50 cells. We further identified miR-124 as a direct regulator of IQGAP1 in EC cells. Enforced expression of miR-124 suppresses EC cell invasion and proliferation. The expression of IQGAP1 mRNA was significantly elevated in EC tissues, while the expression of miR-124 was decreased. The downregulation of miR-124 correlates with a poor survival outcome for patients with EC. Treating EC cells with the demethylating agent 5-aza-2′-deoxycytidine increased miR-124 expression and down-regulated IQGAP1 levels. Our data suggest that IQGAP1 promotes EMT, migration and invasion of EC cells. MiR-124, a novel tumor suppressor miRNA that is epigenetically silenced in EC, can reverse EMT and the invasive properties, by attenuating the expression of the *IQGAP1* oncogene.

## BACKGROUND

The epithelial–mesenchymal transition (EMT), in which polarized epithelial cells become mesenchymal-migrating cells [1], is a key step promoting endometrial cancer (EC) metastasis [2, 3, 4]. IQGAP1 (IQ Motif Containing GTPase Activating Protein 1) is a scaffold protein that directly promotes actin polymerization by binding to F-actin [5], or indirectly modulates the cytoskeleton via interactions with the small GTPases Cdc42 and Rac1 [6]. IQGAP1 is overexpressed in different tumors other than EC, and its upregulation positively correlates with tumor metastasis [7, 8, 9, 10]. IQGAP1 also down-regulates E-cadherin, thereby attenuating cell–cell adhesions and promoting tumor cell invasion [11, 12]. Knocking down IQGAP1 impairs tumor cell growth, migration and invasion and the reversal of the EMT program [13], indicating that IQGAP1 expression is important for the induction of EMT. However, little is known about IQGAP1's expression, biological function and underlying regulatory mechanisms in EC.

MicroRNAs (miRNAs) are small RNA molecules that regulate EMT and metastasis [14]. Other epigenetic mechanisms, such as DNA methylation and histone modification, are critical for the regulation of EMT and miRNA expression in human cancer [15]. DNA methylation-based silencing of tumor suppressive miRNAs, such as miR-34b [16] and miR-124 [17], occurs in various human cancers and stimulates metastasis. IGGAP1 is targeted by miR-124 [18] in hepatocellular carcinoma and by miR-506 in breast cancer [19]. Nevertheless, the contribution of miRNAs to the regulation of IQGAP1 expression in EC is poorly understood.

Here, we provided the first evidence that IQGAP1 functions to promote EMT and invasion in EC cells. Moreover, we uncovered a novel mechanism by which the DNA methylation-associated silence of tumor suppressor miR-124 contributes to the upregulation of IQGAP1, suggesting that targeting the miR-124-IQGAP1 axis may have therapeutic potential for the treatment of invasive ECs.

## RESULTS

### IQGAP1 induces EMT, invasiveness and the proliferation of EC cells

**Figure 1:**
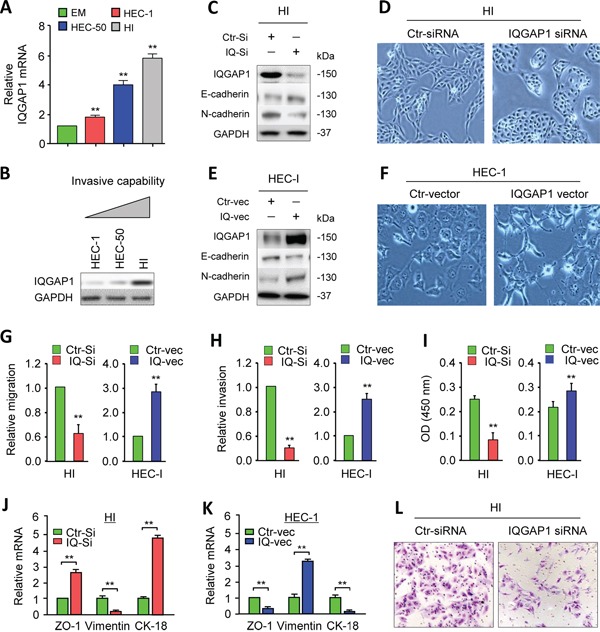
IQGAP1 induces the epithelial-to-mesenchymal transition, invasiveness, and proliferation of endometrial cancer (EC) cells **A.** Reverse transcription quantitative PCR (qPCR) analysis of IQGAP1 mRNA in the immortalized human endometrial cell line EM and the EC cells HEC-1, HEC-50, and HEC-50-HI (HI). The results are presented as the fold-change in expression compared to the EM cells. **B.** Western blot analysis of the IQGAP1 protein in EC cells. **C, E.** The expression of IQGAP1, E-cadherin, and N-cadherin proteins in HI cells transfected with control (Ctr) or IQGAP1 siRNA (C) and in HEC-1 cells expressing either the control or IQGAP1 vector (E). **D, F.** Phase-contrast microscopy shows the morphology of HI cells transfected with control or IQGAP1 siRNA (D) and HEC-1 cells transfected with the control or IQGAP1 vector (F). **G-I.** Detection of migration (G), invasion (H), and proliferation (I) in HI and HEC-1 cells after the indicated transfection. **J, K.** qPCR analysis of *ZO-1*, *CK-18*, and *Vimentin* expression in HI (J) and HEC-1 (K) cells, transfected as indicated. **L.** Representative images from the invasion assays.

Highly invasive subpopulations of HEC-50 cells were previously selected by using a transwell system to create the HEC-50-HI (HI) cell line [3]. We used real-time quantitative, reverse transcription-PCR (qPCR) to gain insight into the roles of IQGAP1 in EC and sought to determine its expression in the immortalized human endometrial epithelial (EM) cells and in three EC cells (HEC-1, HEC-50 and HI). The IQGAP1 mRNA was found to be markedly upregulated in EC cells relative to EM cells (Figure 1A), and its expression level was 3-fold higher in aggressive HI cells than in HEC-1 cells, which have a relatively lower metastatic potential (Figure 1B). This correlation suggests that IQGAP1 might play a role in EC metastasis.

To address this possibility, we used small-interference RNA (siRNA) to transiently knock down IQGAP1 expression in HI cells (Figure 1C) and observed a transition from a mesenchymal morphology to a more epithelial-like shape (Figure 1D), which was accompanied by the loss of N-cadherin expression and gain of E-cadherin expression (Figure 1C). To test whether IQGAP1could induce the EMT program, we further transiently overexpress IQGAP1 in HEC-1 cells (Figure 1E). The ectopic expression of IQGAP1 promoted a mesenchymal morphology (Figure 1F) and resulted in increased N-cadherin and decreased E-cadherin expression (Figure 1E). These data suggest that the IQGAP1 levels are critical for the induction of EMT in EC cells.

To evaluate whether IQGAP1 could modulate the metastatic behavior of EC cells, we performed *in vitro* cell migration, invasion and proliferation assays after the knockdown or overexpression of IQGAP1. Silencing IQGAP1 in HI cells caused a significant decrease in the cell migration, invasion and proliferation, while elevating IQGAP1 expression significantly promoted these characteristics (Figure 1G, 1H, 1I, 1L). At the mRNA level, the qPCR analysis showed that downregulating IQPAP1 in HI cells increased the expression of epithelial markers *ZO-1* and *CK-18*, but reduced the expression of the mesenchymal marker *Vimentin* (Figure 1J). In contrast, the ectopic expression of IQGAP1 in HEC-1 cells activated *Vimentin* expression and inhibited *ZO-1* and *CK-18* levels (Figure 1K). This suggests that IQGAP1 induces mesenchymal-like phenotypes and enhances the mobility, invasion and proliferation of EC cells *in vitro*.

### MiR-124 is down-regulated in highly invasive EC cells and directly suppresses IQGAP1 expression

**Figure 2:**
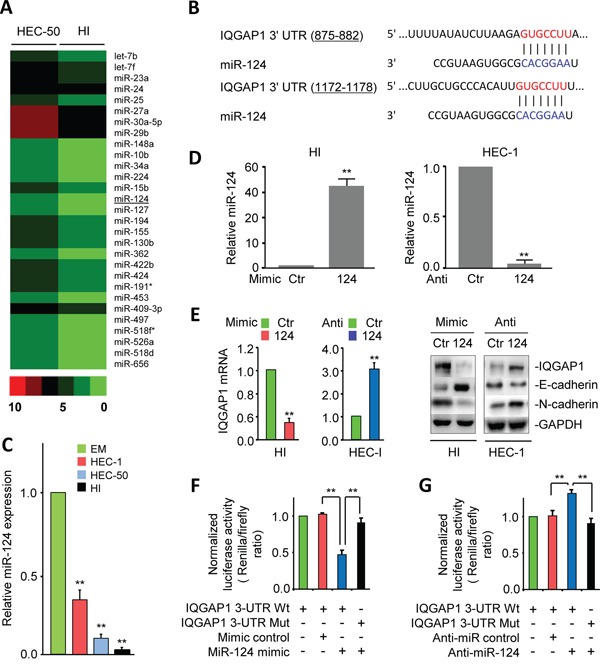
MiR-124 is down-regulated in highly invasive endometrial cancer (EC) cells and directly suppresses IQGAP1 expression **A.** Profiling data of down-regulated microRNAs in highly invasive HEC-50-HI (HI) cells. **B.** Two putative conserved miR-124-binding sites in the *IQGAP1* 3′ untranslated region (3′-UTR). **C.** Relative expression of miR-124 in immortalized human endometrial epithelial and EC cells, assessed by quantitative PCR assays. **D.** Expression of miR-124 in HI or HEC-1 cells transfected with a miR-124 mimic, miR-124 inhibitor, or their respective negative controls. **E.** Expression of the indicated mRNA and proteins in HI and HEC-1 cells after the overexpression or knockdown of miR-124. **F, G.** HI (F) and HEC-1 (G) cells were cotransfected with reporter plasmids containing wild-type *IQGAP1* or a mutant *IQGAP1* 3′-UTR together with a miR-124 mimic, miR-124 inhibitor, or respective negative control. The relative luciferase activity was assayed.

To define the miRNAs that regulate IQGAP1 expression, we profiled the invasive HI and their parental HEC-50 cells using microarray analysis, and identified 29 miRNAs (let-7b, let-7f, miR-10b, miR-15b, miR-23a, miR-24, miR-25, miR-27a, miR-29b, miR-30a-5p, miR-34a, miR-124, miR-127, miR-130b, miR-148a, miR-155, miR-191*, miR-194, miR-224, miR-362, miR-409-3p, miR-422b, miR-424, miR-453, miR-497, miR-518d, miR-518f*, miR-526a and miR-656)that are significantly down-regulated in HI cells compared to HEC-50 cells (Figure 2A). Using qPCR, we validated the down-regulation of these 29 miRNAs in invasive HI cells versus their parental HEC-50 cells (data not shown). Of these miRNAs, we chose to focus on miR-124 because the computational target prediction using TargetScan predicted the presence of two conserved miR-124 seed-matching sequences within the 3′ untranslated region (3′-UTR) of *IQGAP1* mRNA (Figure 2B). To elucidate the relationship between miR-124 and IQGAP1 expression, we examined the expression of miR-124 in different EC cells and found the lowest levels of miR-124 in the highly invasive HI cells (Figure 2C), suggesting that reduced levels of miR-124 cause a dysregulation of IQGAP1 expression. Our qPCR and western blot analyses showed that increasing miR-124 levels in HI cells with the miR-124 mimic reduced IQGAP1 expression, whereas inhibiting miR-124 by means of an anti-miR-124 inhibitor in HEC-1 cells increased IQGAP1 expression (Figure 2D, 2E). In another cellular context, we verified the inhibitory effects of miR-124 on IQGAP1 mRNA expression in human cervical cancer cells (HeLa cells; data not shown). Using a dual-reporter luciferase assay to investigate whether IQGAP1 is directly targeted by miR-124, we found that the IQGAP1 3′-UTR reporter activity was decreased by a miR-124 mimic and increased by the anti-miR-124 inhibitor when the wild-type IQGAP1 3′-UTR was present. However, mutating the miR-124 binding sites in the IQGAP1 3′-UTR completely abrogated these effects (Figure 2F, 2G). Taken together, we showed that the *IQGAP1* mRNA is directly regulated by miR-124 via conserved seed-matching sequences.

### MiR-124 maintains epithelial-like phenotypes and represses cell migration, invasion and proliferation in EC cells

**Figure 3:**
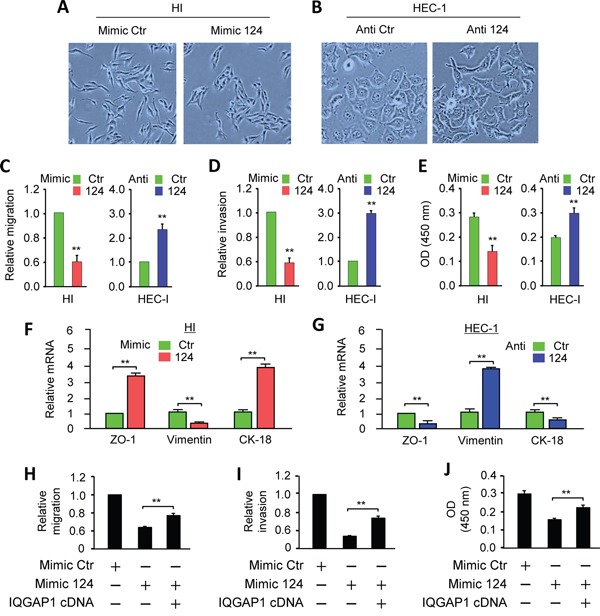
MiR-124 maintains epithelial-like phenotypes and represses cell migration, invasion, and proliferation in endometrial cancer cells **A.** Overexpression of miR-124 in HEC-50-HI (HI) cells with fibroblastic morphology converts them to an epithelial-like morphology. **B.** HEC-1 cells transfected with miR-124 inhibitor exhibit more mesenchymal morphology than the control cells. (A and B, phase-contrast microscopy). **C-E.** Migration (C), invasion (D), and proliferation (E) of HI and HEC-1 cells after the overexpression or knockdown of miR-124. **F, G.** Quantitative PCR analysis of the indicated genes in HI (F) and HEC-1 (G) cells after the overexpression or knockdown of miR-124, as indicated. **H-J.** A miR-124 mimic or its control was transfected into HI cells along with a control vector or the IQGAP1 cDNA vector lacking the 3′-UTR region. The cells were assayed for cell migration (H), invasion (I), and proliferation (J). ^**^ *P* < 0.01.

To determine whether miR-124 inhibits EMT, we evaluated the effects of miR-124 overexpression on the cell morphology and invasion properties. Ectopically expressing miR-124 in HI cells resulted in the occurrence of epithelial morphology (Figure 3A). In contrast, down-regulating of endogenous miR-124 in HEC-1 cells produced a spindle-like morphology (Figure 3B). Cell migration, invasion and proliferation assays demonstrated that miR-124 inhibited the migration, invasion and proliferation of EC cells (Figure 3C, 3D, 3E). Conversely, the loss of miR-124 promoted these malignant features (Figure 3C, 3D, 3E). Furthermore, Western blot and qPCR analyses confirmed that in EC cells, forced expression of miR-124 significantly up-regulated the epithelial markers (E-cadherin, *ZO-1* and *CK-18*), and down-regulated the mesenchymal markers (N-cadherin and *Vimentin*) in EC cells (Figure 2E; Figure 3F, 3G). To examine whether miR-124 suppress oncogenic phenotypes in HI cells through directly down-regulating IQGAP1, we performed the rescue experiments by overexpressing IQGAP1 in HI cells transfected with the miR-124 or a control mimic. Overexpression of IQGAP1 cDNA lacking the 3′-UTR sequence partially restored HI cell migration, invasion and proliferation reduced by miR-124 (Figure 3H, 3I and 3J). These results suggest that miR-124 induces epithelial-like phenotypes, and also indicate that the repression of IQGAP1 by miR-124 represents an important mechanism by which miR-124 suppresses migration, invasion and proliferation.

### Down-regulation of miR-124 is associated with elevated IQGAP1 expression in ECs

**Figure 4:**
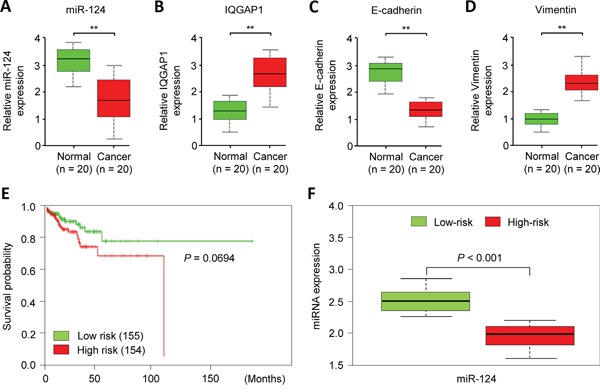
MiR-124 down-regulation is associated with elevated IQGAP1 expression in endometrial cancer cells **A-D.** The expression levels of miR-124 (A), *IQGAP1* (B), *E-cadherin* (C), and *Vimentin* (D) were assessed by a quantitative PCR analysis of 20 paired cancerous and normal tissue samples from endometrial cancer patients. **E.** A Kaplan-Meier survival curve of 309 TCGA (Cancer Genome Atlas database) endometrial cancer samples was created using the SurvMicro database based on the low or high risk for a poor outcome. **F.** Box plots demonstrating significantly lower levels of miR-124 expression in the high-risk patients.

To test whether miR-124/IQGAP1 axis is clinically relevant in EC, we examined tumor specimens and adjacent normal tissues from 20 EC patients. Indeed, the qPCR analysis revealed a negative association between the miR-124 and *IQGAP1* expression levels. Likewise, the expression of miR-124 negatively correlated with the *Vimentin* levels (Figure 4A, 4B, 4D). We also observed a positive correlation between miR-124 and *E-cadherin* expression in the EC tissues (Figure 4A, 4C). To investigate whether the down-regulation of miR-124 is associated with clinical outcomes in EC, we analyze data from 309 EC patients in the Cancer Genome Atlas (TCGA) database by using the SurvMicro web tool [20]. In brief, SurvMicro uses the Cox model and the miRNA levels to give each sample a risk score, and EC patients were stratified into the high-risk (with a low probability of survival; above median of risk score; *n* = 154) or low-risk (with a high probability of survival; below the median of risk score; *n* = 155) group. Kaplan–Meier survival analysis revealed that the overall survival rate in the high-risk group were marginally significantly lower than those in the low-risk group (*P* = 0.0694) and high-risk patients had lower miR-124 expression levels than the low-risk patients (Figure 4E, 4F). Collectively, these data suggest that miR-124 expression is inversely correlated with the IQGAP1 expression level, and decreased miR-124 expression may be associated with poor outcomes for patients with EC. This supports a model that the loss of miR-124 activates IQGAP1 and contributes to EMT and cancer cell invasiveness.

### MiR-124 is epigenetically silenced in EC cells

**Figure 5:**
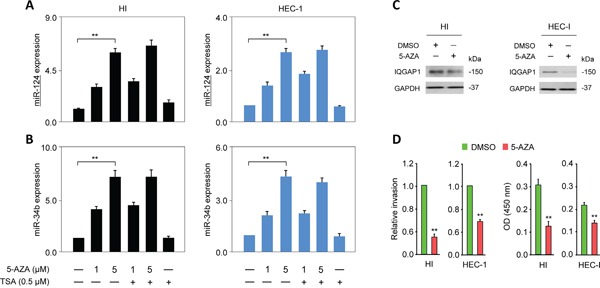
MiR-124 is epigenetically silenced in endometrial cancer cells **A, B.** HI and HEC-1 cells were treated with 5-aza-2′-deoxycytidine (5-AZA), Trichostatin A, or both, after which, quantitative PCR was used to measure the expression levels of miR-124 (A) and miR-34b (B). **C, D.** IQGAP1 protein levels (C), invasion, and proliferation (D) in endometrial cancer cells after treating with 5-AZA.

To examine whether DNA methylation and histone modification could account for the downregulation of miR-124 in EC, we treated EC cells with 5-aza-2′-deoxycytidine (5-AZA; a DNA methylation inhibitor) and/or Trichostatin A (TSA; a histone deacetylase inhibitor). As expected, the tumor suppressor miR-34b was silenced by DNA methylation in the EC cells [16], as shown by its upregulation in the HI and HEC-1 cells treated with 5-AZA. The expression of miR-124 was significantly increased after treating with 5-AZA or a combination of 5-AZA plus TSA (Figure 5A, 5B), but the miR-124 and miR-34b levels remained relatively unchanged in cells treated with TSA alone (Figure 5A, 5B). Treatment with 5-AZA consistently down-regulated the IQGAP1 protein levels (Figure 5C) and markedly reduced cell proliferation and invasion (Figure 5D). These studies suggest that methylation-mediated silencing rather than histone modification serves as an epigenetic event that negatively regulates miR-124 expression. This repression of miR-124 in the EC cells results in the increased abundance of the key oncoprotein IQGAP1 and the subsequent induction of EMT.

## DISCUSSION

IQGAP1 is often overexpressed in many solid tumors (other than EC) and is a key promoter of the EMT program, tumor cell migration, invasion, proliferation and angiogenesis [6-13, 21, 22]. Here we have shown that IQGAP1 induces EMT and enhances EC invasion, and also identified miR-124 as an epigenetically silenced tumor suppressor that inhibits the EC cell migration, invasion and proliferation, by down-regulating oncogene IQGAP1 expression.

The scaffolding protein IQGAP1 functions as a molecular hub for integrating and mediating multiple signaling pathways to promote both tumorigenesis and metastasis [23]. IQGAP1 can affect a wide range of cellular functions by interacting with kinases and other signaling molecules such as Cdc42 [24], Rac1 [25], β-catenin [25], B-Raf [26], extracellular signal-regulated kinase (ERK) [27] and E-cadherin [11]. We show here that IQGAP1 expression is significantly increased in EC tissues. According to our gain- and loss-of-function experiments, IQGAP1 promoted proliferation and induced EMT, which facilitates the migration and invasion of EC cells. Our results provide the first *in vitro* evidence for oncogenic functions of IQGAP1 in EC cells, implying that it could potentially serve as a potential biomarker for the diagnosis and treatment of EC. Interestingly, the disruption of IQGAP1-ERK1/2 interactions with a specific IQGAP1 peptide has been shown to inhibit RAS- and RAF-driven tumorigenesis [28]. Therefore, inhibiting IQGAP1 functions may be a promising targeted therapy for tumors.

Our microarray analysis demonstrated an interesting mRNA expression profile and revealed 29 miRNAs that are significantly down-regulated in highly invasive cells relative to their parental lines. Among these miRNAs, let-7 family members such as let-7f [29], miR-34a [30], miR-127 [31], miR-148a [32], miR-424 [33] and miR-497 [34] are known to display tumor suppressor effects in tumors. In EC, miR-30a-5p expression levels are decreased [35] and the reduced expression of miR-29b correlates with poor disease-free survival [36]. The biological functions and mechanisms of these miRNAs in EC warrant further studies.

MiR-124 exerts tumor suppressor effects and is frequently methylated in multiple cancer types [17]. We demonstrated that miR-124 is down-regulated in EC and the loss of its expression is at least partly mediated by DNA methylation. Overexpressing miR-124 reverses EMT-like phenotypes and reduces EC cell migration, invasion and proliferation. Thus, the restoration of miR-124 by targeted delivery system or by treatment with DNA-demethylating agents may be therapeutically efficacious for the treatment of EC.

In conclusion, our findings provide a new mechanism that accounts for the observed downregulation of miR-124 and upregulation of IQGAP1 in EC. The methylation-mediated repression of miR-124 leads to the overexpression of IQGAP1, which in turn accelerates cancer cell proliferation, EMT and invasion.

## MATERIALS AND METHODS

### Patient samples

After informed consent, 20 pairs of primary ECs and adjacent non-tumor endometrial tissues [37] were collected according to an institutional Review Board-approved protocol at Cancer Center, Sun Yat-Sen University in China. Samples were snap-frozen and stored in liquid nitrogen until the RNA was extracted.

### Cell culture and transfection

The human EC cell lines HEC-1 and HEC-50 were purchased from the JCRB Cell Bank (Osaka. Japan) and cultured in DMEM/F12 medium (Sigma-Aldrich, St. Louis, MO, USA) supplemented with 10% fetal bovine serum (FBS; Invitrogen, Carlsbad, CA, USA). Highly invasive HI cells were established as previously described [3]. The immortalized human endometrial epithelial cell line EM [38] was maintained in DMEM/F12 medium supplemented with 15% FBS. The human cervical cancer cell line, HeLa (ATCC, USA), was grown in DMEM/F12 medium supplemented with 10% FBS. The miR-124 mimic, negative control for the miRNA mimic, anti-miRNA inhibitor for miR-124, negative control for the miRNA inhibitor, IQGAP1 siRNA, and negative control siRNA were all purchased from Ambion (TX, USA). The miRNAs (30 nM) and siRNAs (5 nM) were transiently transfected into cells using Lipofectamine 2000 (Invitrogen, CA, USA) as described by the manufacturer. The pEGFR-IQGAP1-WT vector containing the IQGAP1 cDNA (IQGAP1 vector) and the pEGFR empty control vector, kindly provided by Professor Kozo Kaibuchi (Department of Cell Pharmacology, Nagoya University, Japan), were transiently transfected using the Lipofectamine Plus reagent (Invitrogen, CA, USA). The control vector and a human IQGAP1 cDNA vector lacking the 3′-UTR sequence were obtained from OriGene (MD, USA).

### RNA isolation and qPCR

Total RNA for the microarray and qPCR analyses was extracted using TRIzol (Invitrogen, CA, USA) per the manufacturer protocol. The PrimeScript RT reagent kit (Takara, Japan) was used for the reverse transcription reaction with 100 ng of total RNA. The qPCR gene analysis was performed with Takara SYBR Premix Ex Taq II (Takara, Japan). Primers specific to *IQGAP1* [39] and *GAPDH* [40], which was used as an endogenous control for mRNA expression, have been previously described. Primers for *ZO-1*, *CK-18*, and *Vimentin* were obtained from the PrimerBank database (http://pga.mgh.harvard.edu/primerbank/). miRNA expression was detected by using the NCode miRNA qRT–PCR kit (Invitrogen, CA, USA) according to manufacturer's instructions. The forward primers used for the miRNA quantification were the same exact sequences of the mature miRNA genes. A universal reverse primer was provided in the NCode SYBR miRNA qRT-PCR kit. All miRNA quantification data were normalized to U6 small nuclear RNA [41] and *GAPDH* expression [42] and relative levels were calculated using the 2^−ΔΔCt^ method.

### MiRNA microarray

The microarray analysis was performed using the Superprint G3 Human GE 8 × 60k Microarray (Agilent Technologies) as previously described [43]. The array covers all miRNA transcripts available in the latest version of the Sanger miRBase database. Briefly, total RNA was extracted from EC cells using TRIzol and was purified with the RNeasy MinElute Cleanup kit. MiRNA labeling, hybridization, and washing were performed as described by the manufacturer. Data pre-processing and the differential analysis of the miRNA expression data was done using the AgiMicroRna Bioconductor library. The expression level of miRNAs over 1.5 fold in HI cells with *P* < 0.05 was used as the cutoff to determine the significance compared to their parental HEC-50 cells.

### Western blot

Total protein was collected 48 hours after transfection using the M-Per Mammalian Protein Extraction Reagent (Pierce Biotechnology, MA, USA) as described by the manufacturer. The proteins (40 μg) were separated by SDS-PAGE and transferred to polyvinylidene difluoride membranes for immunoblots with antibodies to IQGAP1 (2C5; Novus Biologicals), E-cadherin (A01589; GenScript), N-cadherin (#610920; BD), and GAPDH (sc-47724; Santa Cruz). These primary antibodies were used at a dilution of 1:1000.

### Cell migration, invasion and proliferation assays

The transwell migration and invasion assay was performed as previously described [3, 4] with transfected cells seeded into the upper chamber (BD Biosciences, MA) with or without a Matrigel coating and DMEM/F12 with 10% FBS in the lower compartment acting as a chemoattractant. Cells were allowed to migrate for 12 and 24 hours in the migration and invasion assays, respectively. The non-motile cells were removed from the top, and the cells in the bottom chamber were stained and counted under a light microscope. Relative migration and invasion activities are expressed as the fold-change over their respective controls. The effect of IQGAP1 or miR-124 on proliferation was measured using Cell Counting Kit-8 (Dojindo, Japan). Briefly, 5 × 103 cells were plated in 96-well plates for 24 hours and then transfected with the IQGAP1 cDNA vector/IQGAP1 siRNA, miR-124 mimics/inhibitors, and their respective controls. At 72 hours, the absorbance of the cells was measured with a spectrophotometer at 450 nm.

### Luciferase activity assay

The Renilla luciferase-reporter plasmids containing human *IQGAP1* mRNA [44] with either wild-type (Wt; #14503) or mutant (Mut; #14504) miR-124 binding sites (875–882 bp and 1172–1178 bp from the start site of the 3′-UTR) were obtained from Addgene (Cambridge, MA, USA). Wt or Mut IQGAP1 reporter vectors, together with the pGL3-basic firefly luciferase expression vector as a reference control (Promega, San Luis Obispo, CA, USA), were transfected with 30 nM of the miR-124 mimic or inhibitor using Lipofectamine 2000 (Invitrogen, CA, USA). The Renilla and firefly luciferase activities were measured using the Dual Luciferase assay kit (Promega, WI, USA) 24 hours after transfection. The Renilla luciferase activity was normalized to the firefly luciferase activity.

### Drug treatment

Cells were treated with 5-AZA (1 or 5 μM; Sigma-Aldrich) for 72 hours or TSA (0.5 μM; Sigma-Aldrich) for 24 hours. For the combination study, the 5-AZA (5 μM) was present for 72 hours and the TSA (0.5 μM) was added for the last 24 hours. The media were changed daily, and fresh drug was added.

### Statistical analysis

Data are presented as the mean ± SEM of at least three independent experiments performed in triplicate. If not specified otherwise, the experimental values are expressed as fold-changes normalized to their respective controls. Statistical significance was assessed by the two-tailed Student t-test (*P < 0.05; **P < 0.01). The differences between the cancer and normal tissues were analyzed using the Wilcoxon matched-pairs test.
